# Atrophy of White Adipose Tissue Accompanied with Decreased Insulin-Stimulated Glucose Uptake in Mice Lacking the Small GTPase Rac1 Specifically in Adipocytes

**DOI:** 10.3390/ijms221910753

**Published:** 2021-10-05

**Authors:** Kiko Hasegawa, Nobuyuki Takenaka, Kenya Tanida, Man Piu Chan, Mizuki Sakata, Atsu Aiba, Takaya Satoh

**Affiliations:** 1 Laboratory of Cell Biology, Department of Biological Science, Graduate School of Science, Osaka Prefecture University, Sakai, Osaka 599-8531, Japan; kikohasegawa18@b.s.osakafu-u.ac.jp (K.H.); takenaka@b.s.osakafu-u.ac.jp (N.T.); ktanida21@gmail.com (K.T.); md304019@edu.osakafu-u.ac.jp (M.P.C.); mizuki20@b.s.osakafu-u.ac.jp (M.S.); 2 Laboratory of Animal Resources, Center for Disease Biology and Integrative Medicine, Graduate School of Medicine, The University of Tokyo, Bunkyo-ku, Tokyo 113-0033, Japan; aiba@m.u-tokyo.ac.jp

**Keywords:** Akt2, glucose uptake, GLUT4, GTPase, insulin, phosphoinositide 3-kinase, Rac1, RalA, white adipose tissue

## Abstract

Insulin stimulates glucose uptake in adipose tissue and skeletal muscle by inducing plasma membrane translocation of the glucose transporter GLUT4. Although the small GTPase Rac1 is a key regulator downstream of phosphoinositide 3-kinase (PI3K) and the protein kinase Akt2 in skeletal muscle, it remains unclear whether Rac1 also regulates glucose uptake in white adipocytes. Herein, we investigated the physiological role of Rac1 in white adipocytes by employing adipocyte-specific *rac1* knockout (adipo-*rac1*-KO) mice. Subcutaneous and epididymal white adipose tissues (WATs) in adipo-*rac1*-KO mice showed significant reductions in size and weight. Actually, white adipocytes lacking Rac1 were smaller than controls. Insulin-stimulated glucose uptake and GLUT4 translocation were abrogated in *rac1*-KO white adipocytes. On the other hand, GLUT4 translocation was augmented by constitutively activated PI3K or Akt2 in control, but not in *rac1*-KO, white adipocytes. Similarly, to skeletal muscle, the involvement of another small GTPase RalA downstream of Rac1 was demonstrated. In addition, mRNA levels of various lipogenic enzymes were down-regulated in *rac1*-KO white adipocytes. Collectively, these results suggest that Rac1 is implicated in insulin-dependent glucose uptake and lipogenesis in white adipocytes, and reduced insulin responsiveness due to the deficiency of Rac1 may be a likely explanation for atrophy of WATs.

## 1. Introduction

Two major types of adipose tissue, white adipose tissue (WAT) and brown adipose tissue (BAT), are known in mammals. White adipocytes store fat, mainly triglycerides, in one large lipid droplet, whereas brown adipocytes contain a large number of small lipid droplets and mitochondria, generating heat to maintain body temperature. In adipose tissue, insulin stimulates the uptake of glucose and fatty acids from the blood, enhances fatty acid and triglyceride synthesis, and decreases the rate of lipolysis. Thus, insulin exerts an anabolic effect on adipose tissue [1].

The glucose transporter GLUT4 that is localized in the plasma membrane mediates the insulin-stimulated glucose transport in skeletal muscle and adipose tissue [2,3]. In unstimulated cells, GLUT4 molecules are sequestered in specialized intracellular compartments, termed GLUT4 storage vesicles. Following insulin stimulation, intracellular trafficking of vesicles containing GLUT4 and their fusion with the plasma membrane are stimulated. Consequently, GLUT4 is redistributed to the plasma membrane, enhancing glucose uptake.

The intracellular signaling mechanisms underlying insulin-stimulated GLUT4 translocation to the plasma membrane are thought to be conserved, at least in part, between skeletal muscle and adipose tissue [3,4,5,6]. A kinase cascade consisting of phosphoinositide 3-kinase (PI3K) and serine/threonine protein kinases, PDK1 and Akt2, plays a critical role downstream of the insulin receptor in both skeletal muscle and adipose tissue. A variety of substrate proteins of Akt2, including the Akt substrate of 160 kDa (AS160, also termed TBC1D4) [7], are implicated as regulators of GLUT4 vesicle trafficking. AS160 acts as a GTPase-activating protein (GAP) for Rab family small GTPases, Rab8A and Rab13, in skeletal muscle cells [8] and Rab10 in adipocytes [9,10], regulating the activity of Rab proteins in response to insulin stimulation. On the other hand, a signaling pathway composed of the Cbl proto-oncogene product, adaptor proteins CAP and CrkII, the guanine nucleotide exchange factor (GEF) C3G, and the Rho family small GTPase TC10 is known to be a PI3K-independent pathway that is unique to adipocytes [11].

The Rho family small GTPase Rac1 is also implicated in insulin-stimulated glucose uptake in skeletal muscle cells [3,5,6,12,13,14,15,16,17]. The involvement of Rac1 in insulin-stimulated GLUT4 translocation was initially suggested in cultured myoblasts and in vitro differentiated myotubes [12,13,14,16], and was further supported by studies using mouse skeletal muscle [15,17]. Moreover, muscle-specific *rac1* knockout mice actually showed impaired glucose tolerance and higher plasma insulin concentrations after intraperitoneal glucose injection, providing evidence that Rac1 plays a physiologically important role in insulin-dependent glucose uptake in skeletal muscle [15].

The mechanisms by which Rac1 is regulated in response to insulin stimulation have been studied using myoblast cell lines and mouse skeletal muscle. We showed that ectopic expression of a constitutively activated mutant of PI3K or Akt2 caused Rac1 activation in L6 myoblasts and mouse gastrocnemius muscle fibers [18,19,20]. Moreover, these constitutively activated mutants induced translocation of GLUT4 to the plasma membrane in wild-type, but not in *rac1*-KO, mouse gastrocnemius muscle fibers [19]. Taken together, we propose that Rac1 lies downstream of Akt2, regulating insulin signaling. On the other hand, it is also proposed that Rac1 functions downstream of PI3K, but not Akt2, and Akt2 and Rac1 are responsible for exocytosis of GLUT4-containing vesicles and cytoskeletal rearrangements, respectively [5,6,21,22].

Given that the signaling mechanisms mediated by PI3K and Akt2 for insulin-stimulated glucose uptake are conserved between skeletal muscle and adipose tissue, it is possible that Rac1 serves as a molecular switch of insulin-stimulated glucose uptake downstream of PI3K and Akt2, not only in skeletal muscle but also in adipose tissue. Consistent with this idea, we demonstrated that Rac1 was indeed activated in a PI3K-dependent manner following in vitro insulin stimulation of primary cultured mouse adipocytes [23]. Similar results were also obtained in adipose tissue of living mice after intravenous injection of insulin [23]. Furthermore, a critical role of Rac1, downstream of Akt2 and upstream of the Ras family GTPase RalA in insulin signaling, was demonstrated in differentiated mouse 3T3-L1 adipocytes [24]. The GEF FLJ00068, which was identified as the GEF for Rac1 in skeletal muscle insulin signaling [16,25], was recently reported to be responsible for Rac1 activation in 3T3-L1 adipocytes as well [26]. In marked contrast to the above results, a previous study by Marcusohn et al. argued against the involvement of Rac1 in insulin regulation of glucose transport based on the results obtained from analyses using 3T3-L1 adipocytes [27]. To resolve this controversy about whether Rac1 is implicated in adipocyte insulin signaling for glucose uptake, we further investigated the role of Rac1, employing *rac1*-KO mouse white adipocytes in this study.

## 2. Results

### 2.1. Atrophy of WAT in Adipocyte-Specific Rac1 Knockout (Adipo-Rac1-KO) Mice

**Figure 1:**
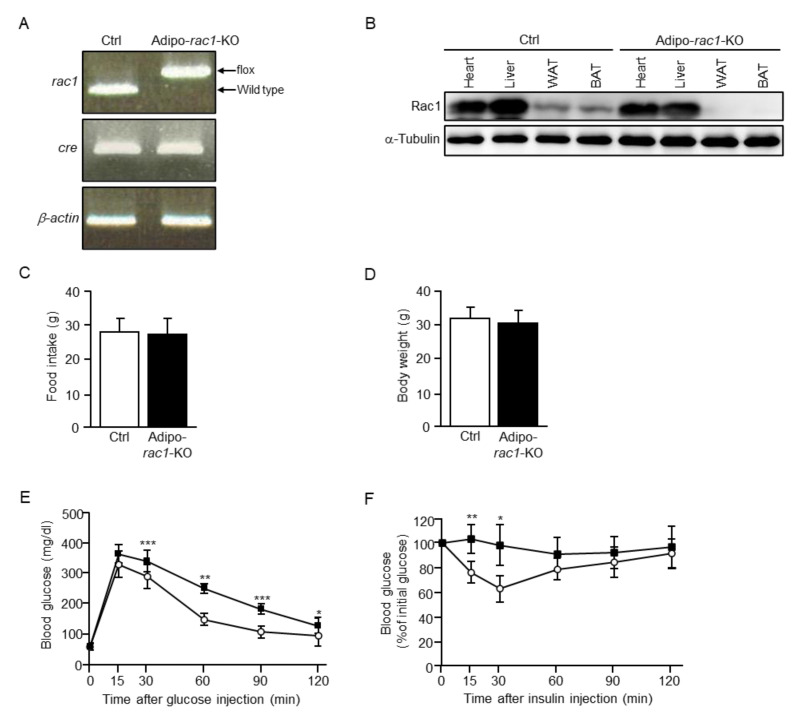
The generation of adipo-*rac1*-KO mice. (**A**) Genotyping of control (Ctrl, Adipoq-Cre transgenic) and adipo-*rac1*-KO (*rac1^flox/flox^*; Adipoq-Cre) mice. Positions of polymerase chain reaction (PCR) products from wild-type and floxed alleles of the *rac1* gene are indicated. (**B**) Expression of Rac1 and α-tubulin (loading control) in various tissues of control and adipo-*rac1*-KO mice. Rac1 and α-tubulin were visualized by immunoblot analysis using anti-Rac1 and anti-α-tubulin antibodies, respectively. (**C**) Food intake (per week) in 26-week-old control and adipo-*rac1*-KO mice. Data are shown as means ± S.E. (*n* = 10). (**D**) Body weight in 26-week-old control and adipo-*rac1*-KO mice. Data are shown as means ± S.E. (*n* = 10). (**E**) Intraperitoneal glucose tolerance test in 26-week-old control (white circle) and adipo-*rac1*-KO (black square) mice. Data are shown as means ± S.E. (*n* = 5). * *p* < 0.05, ** *p* < 0.01, *** *p* < 0.001. (**F**) Intraperitoneal insulin tolerance test in 26-week-old control (white circles) and adipo-*rac1*-KO (black squares) mice. Data are shown as means ± S.E. (*n* = 5). * *p* < 0.05, ** *p* < 0.01.

*Rac1^flox/flox^* mice [28] were crossed with Adipoq-Cre transgenic mice, in which Cre recombinase was expressed in a highly adipocyte-specific manner [29], to generate adipo-*rac1*-KO (*rac1^flox/flox^*; Adipoq-Cre) mice. Adipoq-Cre transgenic mice were used as controls throughout this study (Figure 1A). Adipo-*rac1*-KO mice were born without any intrauterine loss or early death as suggested by the generation of *rac1^flox/flox^* and adipo-*rac1*-KO littermates at the expected Mendelian ratios after the crossing of *rac1^flox/flox^* mice with adipo-*rac1*-KO mice (data not shown). Immunoblot analysis revealed the complete absence of the Rac1 protein in WAT and BAT, with intact expression in the heart and liver (Figure 1B).

Adipo-*rac1*-KO mice were indistinguishable from their *rac1^flox/flox^* littermates in appearance and behavior, and both male and female adipo-*rac1*-KO mice were fertile. In addition, no significant reduction in food intake and body weight was observed in adipo-*rac1*-KO mice (Figure 1C,D). Notably, intraperitoneal glucose and insulin tolerance tests demonstrated that adipo-*rac1*-KO mice developed whole-body glucose intolerance and insulin resistance (Figure 1E,F).

**Figure 2:**
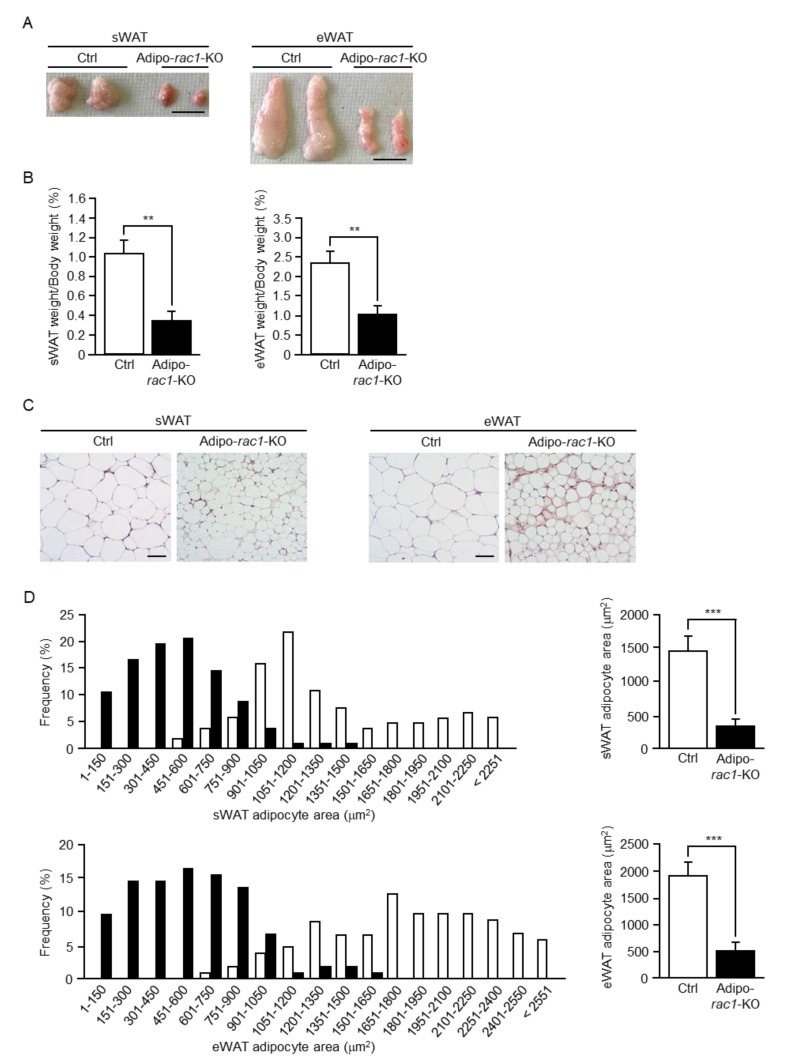
Atrophy of WAT in adipo-*rac1*-KO mice. (**A**) Representative images of sWAT and eWAT excised from 26-week-old control and adipo-*rac1*-KO mice. Scale bar, 1 cm. (**B**) Wet weight (relative to body weight) of sWAT and eWAT excised from 26-week-old control and adipo-*rac1*-KO mice. Data are shown as means ± S.E. (*n* = 10). ** *p* < 0.01. (**C**) Hematoxylin-eosin staining of sWAT and eWAT sections prepared from 26-week-old control and adipo-*rac1*-KO mice. Scale bar, 50 μm. (**D**) Adipocyte areas of sWAT and eWAT excised from 26-week-old control and adipo-*rac1*-KO mice. Histograms depict the distribution of areas of control (white bars) and *rac1*-KO (black bars) white adipocytes. Data are shown as means ± S.E. (*n* = 100). *** *p* < 0.001.

Anatomical and microscopic examinations revealed a significant difference between control and *rac1*-KO WATs in size and weight. Both subcutaneous white adipose tissue (sWAT) and epididymal white adipose tissue (eWAT) excised from adipo-*rac1*-KO mice were significantly smaller than those from control mice, suggesting severe atrophy of both sWAT and eWAT in adipo-*rac1*-KO mice at the age of 26 weeks (Figure 2A,B). Correspondingly, the size of each adipocyte in sWAT and eWAT was largely reduced in adipo-*rac1*-KO mice compared to those in control mice (Figure 2C,D).

### 2.2. Effect of Rac1 KO on Glucose Uptake and GLUT4 Translocation Induced by Insulin or a Constitutively Activated Mutant of PI3K, Akt2, or FLJ00068

**Figure 3:**
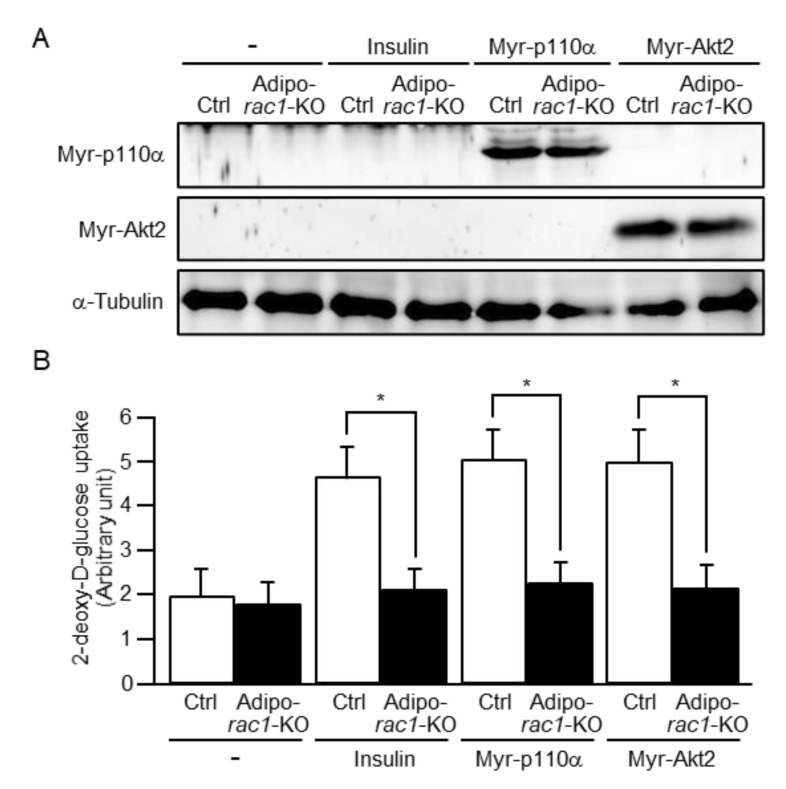
Inhibition of glucose uptake induced by insulin or a constitutively activated mutant of PI3K or Akt2 in white adipocytes from adipo-*rac1*-KO mice. (**A**) Ectopically expressed Myr-p110α and Myr-Akt2 were detected by immunoblot analysis using an anti-hemagglutinin (HA) tag antibody. α-Tubulin (loading control) was detected by immunoblot analysis using an anti-α-tubulin antibody. (**B**) Uptake of 2-DG induced by insulin or a constitutively activated mutant of PI3K or Akt2 in white adipocytes from control (Ctrl) and adipo-*rac1*-KO mice was measured. Data are shown as means ± S.E. (*n* = 5). * *p* < 0.05.

As an approach to reveal the mechanisms underlying atrophy of WAT, glucose uptake in primary cultured white adipocytes was examined. An N-terminally myristoylated form of the phosphoinositide 3-kinase catalytic subunit p110α (Myr-p110α) and an N-terminally myristoylated form of Akt2 (Myr-Akt2) are known as constitutively activated mutants of PI3K and Akt2, respectively. These mutants were ectopically expressed in mouse sWAT via in vivo electroporation (Figure 3A). Serine phosphorylation of the hydrophobic motif in the C-terminal portion of Akt2 was observed following ectopic expression of Myr-p110α in both control and adipo-*rac1*-KO mice (data not shown). Adipocytes in sWAT that was ablated from the above-described mice were then cultivated. In some experiments, cells were stimulated by insulin. Subsequently, glucose uptake in these primary cultured white adipocytes was measured. Insulin stimulation and ectopic expression of the above constitutively activated mutants significantly enhanced the uptake of 2-deoxy-D-glucose (2-DG) in white adipocytes from control mice (Figure 3B). In marked contrast, these stimulations failed to enhance glucose uptake in white adipocytes from adipo-*rac1*-KO mice (Figure 3B).

**Figure 4:**
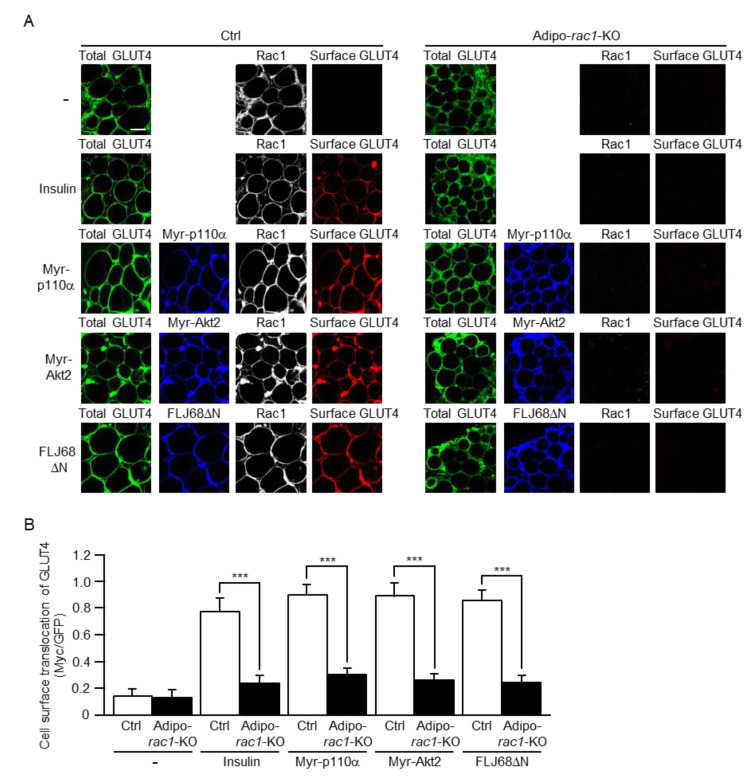
Inhibition of cell surface translocation of GLUT4 induced by insulin or a constitutively activated mutant of PI3K, Akt2, or FLJ00068 in *rac1*-KO sWAT. (**A**) The total amount of GLUT4*myc*7-GFP was estimated by the fluorescent intensity of GFP. Myr-p110α, Myr-Akt2, and FLJ68ΔN were visualized by immunofluorescent microscopy using an anti-HA antibody. Cell surface-localized GLUT4*myc*7-GFP was visualized by immunofluorescent microscopy using an anti-Myc antibody. Scale bar, 50 μm. (**B**) Cell surface translocation of GLUT4*myc*7-GFP shown in (**A**) was quantified. Data are shown as means ± S.E. (*n* = 15). *** *p* < 0.001.

Glucose uptake occurs via translocation of GLUT4 from intracellular storage sites to the plasma membrane in white adipocytes. Therefore, we next examined GLUT4 translocation by employing a GLUT4 reporter containing a green fluorescent protein (GFP) and exofacial Myc tags (GLUT4*myc*7-GFP) [30]. GLUT4*myc*7-GFP was ectopically expressed in mouse sWAT in combination with either one of Myr-p110α, Myr-Akt2, and FLJ68ΔN via in vivo electroporation. FLJ68ΔN is a constitutively activated mutant of the GEF FLJ00068, which is responsible for the insulin-dependent activation of Rac1 in 3T3-L1 adipocytes [26]. Insulin was administered via intravenous injection, and sWAT was ablated from stimulated and unstimulated mice. Isolated sWAT was then subjected to immunofluorescent microscopy. The expression level (total amount) of GLUT4*myc*7-GFP in the cell was estimated by the fluorescent intensity of GFP. GLUT4*myc*7-GFP localized in the plasma membrane was monitored by immunofluorescent microscopy using an antibody against the exofacial Myc tag. Cell surface translocation of GLUT4 was induced by insulin stimulation and ectopic expression of constitutively activated mutants of PI3K, Akt2, and FLJ00068 in control mice (Figure 4). Stimulation-dependent GLUT4 translocation in vivo, as observed in control mice, was remarkably suppressed in adipo-*rac1*-KO mice (Figure 4). These results are in good agreement with those of 2-DG uptake (Figure 3).

### 2.3. The Activation of Rac1 by Insulin or a Constitutively Activated Mutant of PI3K, Akt2, or FLJ00068 and the Effect of an Akt2-Specific Inhibitor on Rac1 Activation

**Figure 5:**
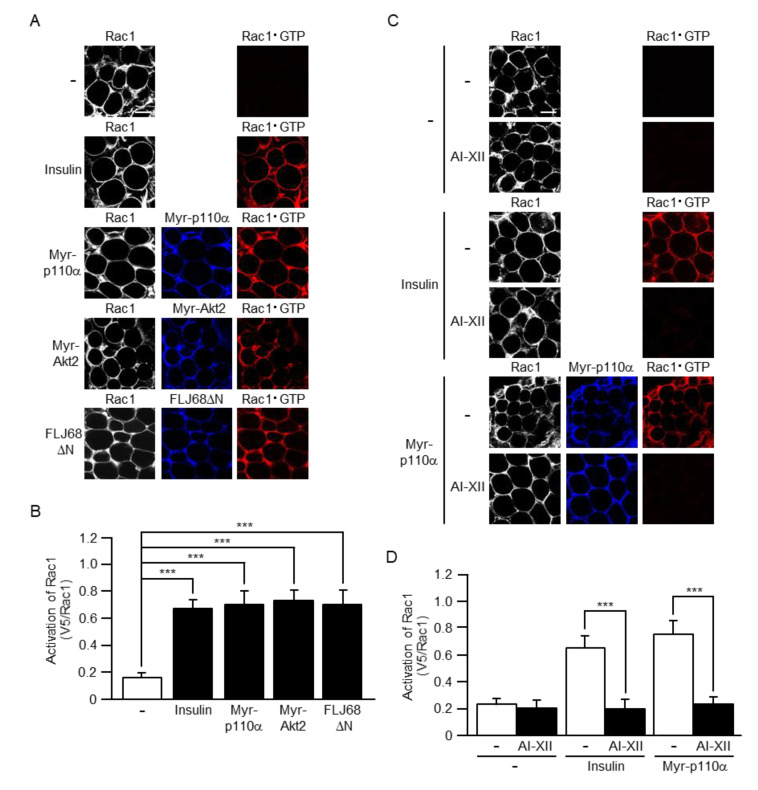
The activation of Rac1 by insulin or a constitutively activated mutant of PI3K, Akt2, or FLJ00068 and the effect of an Akt2-specific inhibitor on Rac1 activation. (**A**) Endogenous Rac1 was visualized by immunofluorescent microscopy using an anti-Rac1 antibody. Myr-p110α, Myr-Akt2, and FLJ68ΔN were visualized by immunofluorescent microscopy using an anti-HA antibody. Rac1·GTP was visualized by immunofluorescent microscopy using the activation-specific probe GST-POSH(251–489)-V5×3 and an anti-V5 antibody. Scale bar, 50 μm. (**B**) The activation of Rac1 shown in (**A**) was quantified. Data are shown as means ± S.E. (*n* = 15). *** *p* < 0.001. (**C**) Endogenous Rac1 was visualized by immunofluorescent microscopy using an anti-Rac1 antibody. Myr-p110α was visualized by immunofluorescent microscopy using an anti-HA antibody. Rac1·GTP was visualized by immunofluorescent microscopy using the activation-specific probe GST-POSH(251–489)-V5×3 and an anti-V5 antibody. In some experiments, excised sWAT was treated with AI-XII (5 µM) for 2 h. Scale bar, 50 µm. (**D**) The activation of Rac1 shown in (**C**) was quantified. Data are shown as means ± S.E. (*n* = 15). *** *p* < 0.001.

Although we previously demonstrated that, in 3T3-L1 adipocytes, ectopic expression of a constitutively activated mutant of Akt2 or FLJ00068 induced the activation of Rac1 [24,26], it remains unclear whether these signaling proteins could activate Rac1 in white adipocytes in vivo. Therefore, we next examined the activation of Rac1 following ectopic expression of Myr-p110α, Myr-Akt2, or FLJ68ΔN in mouse sWAT via in vivo electroporation. The activated form of Rac1 (Rac1·GTP) was detected by the specific polypeptide probe glutathione S-transferase (GST)-POSH(251–489)-V5×3, which was subsequently visualized by immunofluorescent microscopy using an antibody against the V5 tag. Intravenously administrated insulin actually induced Rac1 activation as previously described [23]. Furthermore, all of the constitutively activated mutants tested activated Rac1 to a similar extent as in the case of insulin (Figure 5A,B).

To further confirm the hypothesis that Akt2 is involved in Rac1 activation downstream of PI3K, the effect of an Akt2-specific inhibitor was then tested. Following ectopic expression of Myr-p110α in mouse white adipocytes, sWAT was excised, and subsequently subjected to treatment with the Akt2-specific inhibitor AI-XII. In this experiment, sWAT was stimulated by insulin ex vivo after treatment with AI-XII. AI-XII almost completely abolished the activation of Rac1 following insulin stimulation or ectopic expression of Myr-p110α (Figure 5C,D). Therefore, it is plausible that Akt2 plays a pivotal role in insulin-stimulated Rac1 activation in white adipocytes.

### 2.4. Effect of a Dominant-Negative RalA Mutant on GLUT4 Translocation Induced by Insulin or a Constitutively Activated Mutant of PI3K, Akt2, FLJ00068, or Rac1

RalA is a member of the Ras family of small GTPases, regulating GLUT4 translocation in adipocytes and skeletal muscle [23,31,32,33]. In skeletal muscle insulin signaling, RalA was reported to serve as a regulator of GLUT4 translocation downstream of Rac1 [32,33]. Additionally, the involvement of RalA downstream of Rac1 was recently demonstrated in in vitro differentiated 3T3-L1 adipocytes [24]. However, in vivo compelling evidence for this was lacking, and therefore, we attempted to examine whether RalA played a pivotal role downstream of Rac1 in mouse white adipocytes.

**Figure 6:**
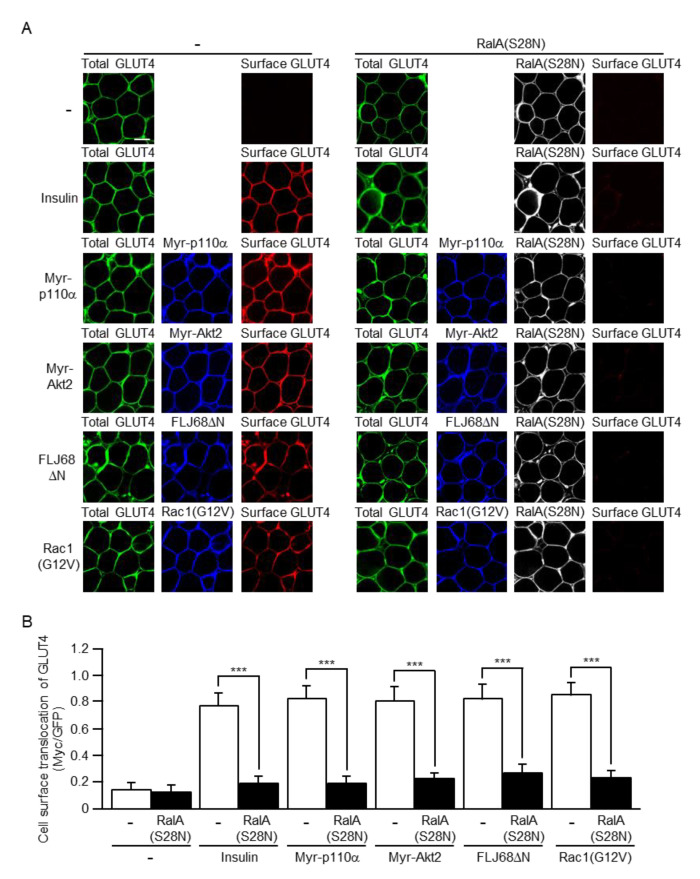
Inhibition of cell surface translocation of GLUT4 induced by insulin or a constitutively activated mutant of PI3K, Akt2, FLJ00068, or Rac1 by a dominant-negative mutant of RalA. (**A**) The total amount of GLUT4*myc*7-GFP was estimated by the fluorescent intensity of GFP. Myr-p110α, Myr-Akt2, FLJ68ΔN, and Rac1(G12V) were visualized by immunofluorescent microscopy using an anti-HA antibody. Cell surface-localized GLUT4*myc*7-GFP was visualized by immunofluorescent microscopy using an anti-Myc antibody. Scale bar, 50 μm. (**B**) Cell surface translocation of GLUT4*myc*7-GFP shown in (**A**) was quantified. Data are shown as means ± S.E. (*n* = 15). *** *p* < 0.001.

RalA(S28N) is widely known as a dominant-negative mutant. In fact, ectopically expressed RalA(S28N) significantly inhibited GLUT4 translocation in response to insulin or constitutively activated mutants of Rac1 and its upstream regulators in skeletal muscle [33]. Thus, we employed this dominant-negative mutant to investigate the involvement of RalA downstream of Rac1 in insulin-stimulated GLUT4 translocation in white adipocytes in vivo. GLUT4*myc*7-GFP was ectopically expressed in mouse sWAT in combination with either one of constitutively activated mutants via in vivo electroporation. In some experiments, the dominant-negative mutant RalA(S28N) was also ectopically expressed. Insulin was administered via intravenous injection. Following this, sWAT was ablated from stimulated and unstimulated mice, and then subjected to immunofluorescent microscopy. Actually, cell surface translocation of GLUT4 induced by insulin or any one of the constitutively activated mutants, including Myr-p110α, Myr-Akt2, FLJ68ΔN, and Rac1(G12V), was totally inhibited by the dominant-negative mutant RalA(S28N) (Figure 6). These results are fully consistent with those obtained from our previous experiments by the use of 3T3-L1 adipocytes [24].

### 2.5. Effect of Rac1 KO on the Activation of RalA Induced by Insulin or a Constitutively Activated Mutant of PI3K, Akt2, FLJ00068, or Rac1

**Figure 7:**
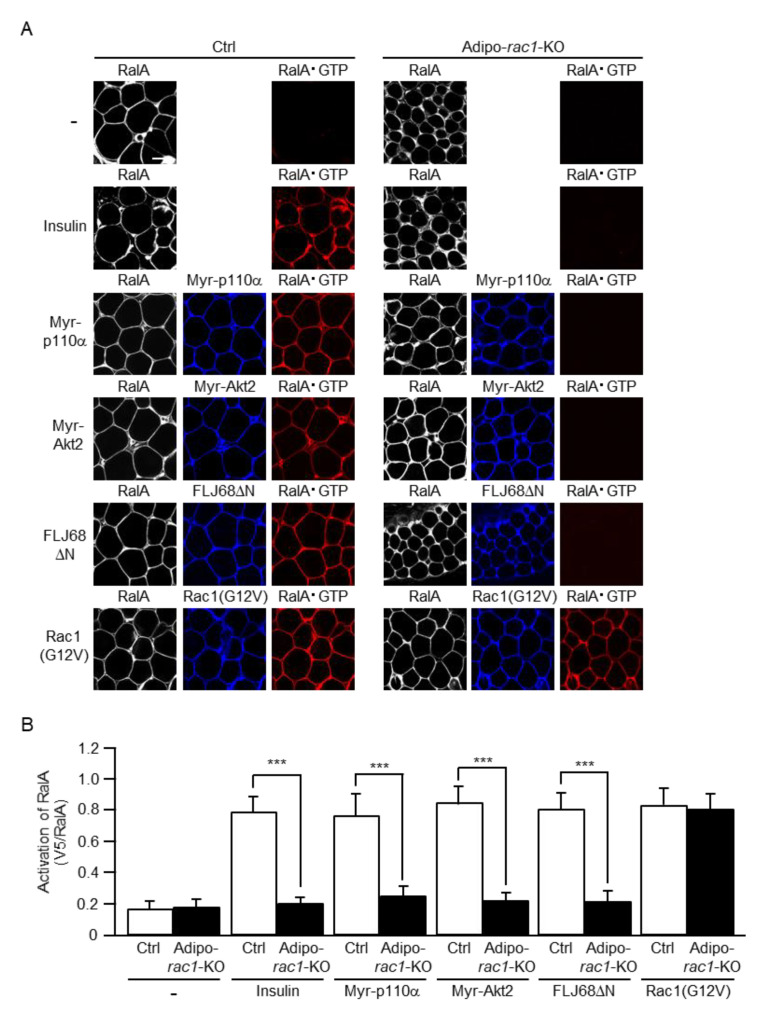
Inhibition of the activation of RalA induced by insulin or a constitutively activated mutant of PI3K, Akt2, or FLJ00068 in *rac1*-KO sWAT. (**A**) Endogenous RalA was visualized by immunofluorescent microscopy using an anti-RalA antibody. Myr-p110α, Myr-Akt2, FLJ68ΔN, and Rac1(G12V) were visualized by immunofluorescent microscopy using an anti-HA antibody. RalA·GTP was visualized by immunofluorescent microscopy using the activation-specific probe GST-V5×3-Sec5(1–99) and an anti-V5 antibody. Scale bar, 50 μm. (**B**) The activation of RalA shown in (**A**) was quantified. Data are shown as means ± S.E. (*n* = 15). *** *p* < 0.001.

It is important to examine the effect of *rac1* KO on the activation of RalA in response to various upstream signals to confirm the involvement of Rac1 upstream of RalA in vivo. Myr-p110α, Myr-Akt2, FLJ68ΔN, and Rac1(G12V) were ectopically expressed in mouse sWAT via in vivo electroporation. The activated form of RalA (RalA·GTP) was detected by the specific polypeptide probe GST-V5×3-Sec5(1–99), which was subsequently visualized by immunofluorescent microscopy using an antibody against the V5 tag. Insulin, when intravenously administrated, induced RalA activation as previously described [23]. Furthermore, all of the constitutively activated mutants tested activated RalA to a similar extent, as in the case of insulin (Figure 7). Notably, in adipo-*rac1*-KO mice, the insulin-dependent activation of RalA in white adipocytes was almost completely prevented (Figure 7). Moreover, RalA activation following ectopic expression of any one of the constitutively activated mutants of signaling components upstream of Rac1 was totally suppressed in *rac1*-KO mouse white adipocytes (Figure 7). Taken together, these results strongly support the idea that Rac1 is critically involved in the regulation of RalA in white adipocyte insulin signaling.

### 2.6. Effect of Akt2- and Rac1-Specific Inhibitors on the Activation of RalA Induced by Insulin or a Constitutively Activated Mutant of PI3K

**Figure 8:**
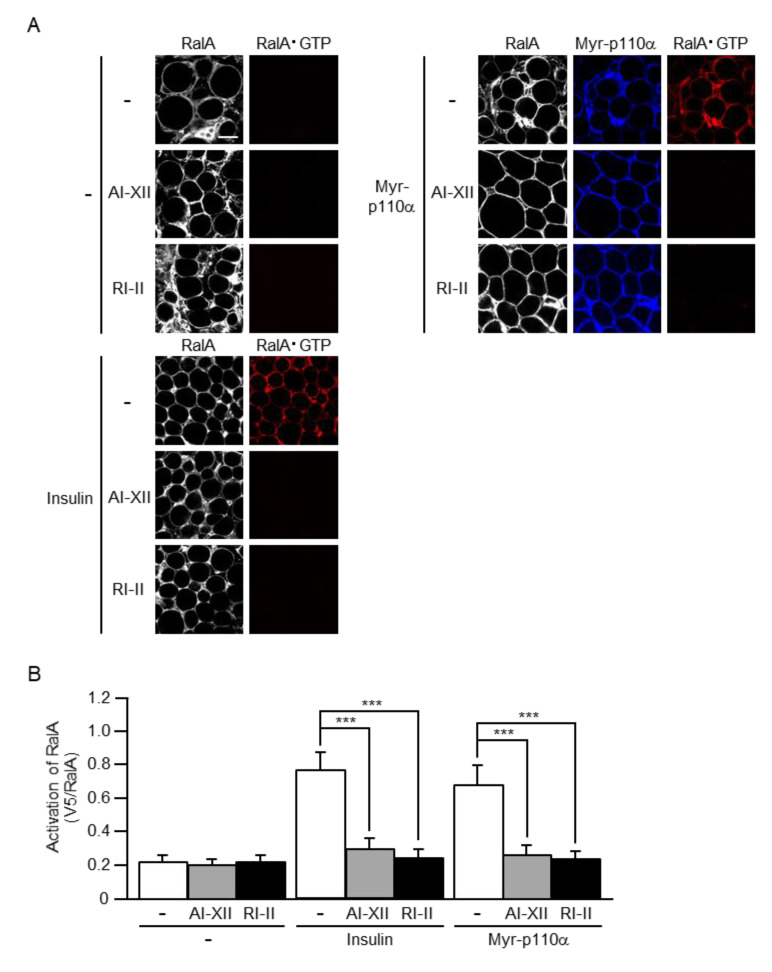
Inhibition of the activation of RalA induced by insulin or a constitutively activated mutant of PI3K by Akt2- and Rac1-specific inhibitors. (**A**) Endogenous RalA was visualized by immunofluorescent microscopy using an anti-RalA antibody. Myr-p110α was visualized by immunofluorescent microscopy using an anti-HA antibody. RalA·GTP was visualized by immunofluorescent microscopy using the activation-specific probe GST-V5×3-Sec5(1–99) and an anti-V5 antibody. In some experiments, excised sWAT was treated with AI-XII (5 µM) or RI-II (25 µM) for 2 h. Scale bar, 50 µm. (**B**) The activation of RalA shown in (**A**) was quantified. Data are shown as means ± S.E. (*n* = 15). *** *p* < 0.001.

To further confirm the hypothesis that RalA acts downstream of Akt2 and Rac1, we next tested the effect of Akt2- and Rac1-specific inhibitors on RalA activation by insulin or a constitutively activated mutant of PI3K. Myr-p110α was ectopically expressed in mouse sWAT, which was then excised and subjected to treatment with the Akt2-specific inhibitor AI-XII or the Rac1-specific inhibitor RI-II. In this experiment, sWAT was stimulated by insulin ex vivo after treatment with AI-XII or RI-II. Both AI-XII and RI-II almost completely abolished the activation of RalA by insulin or Myr-p110α (Figure 8). These results support the notion that RalA is regulated downstream of Akt2 and Rac1 in white adipocytes, as well as in skeletal muscle.

### 2.7. Effect of Rac1 KO on mRNA Levels of Various Lipogenic Enzymes

**Figure 9:**
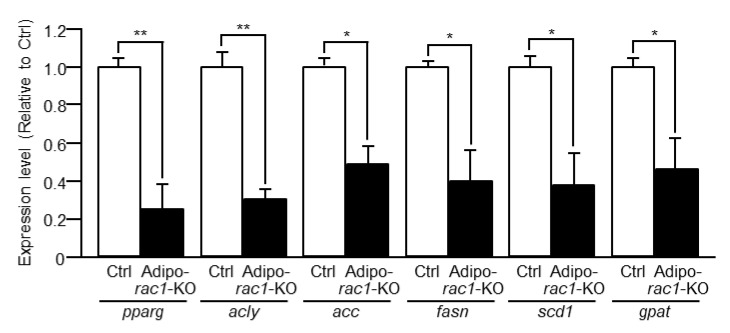
Reduced expression of genes encoding lipogenic enzymes in *rac1*-KO sWAT. Relative expression levels in *rac1*-KO sWAT were determined by quantitative RT-PCR analysis. Data are shown as means ± S.E. (*n* = 6). * *p* < 0.05, ** *p* < 0.01.

To explore the possibility that Rac1 regulates not only the induction of glucose uptake but also other adipocyte functions, such as de novo lipid synthesis, we next examined expression levels of various genes involved in adipocyte functions by quantitative reverse transcriptase (RT)-PCR analysis. Peroxisome proliferator-activated receptor γ (PPARγ) is known to act as a key nuclear receptor in adipocyte differentiation and function through the activation of various target genes [34]. The expression level of the *pparg* gene, which encodes PPARγ, significantly decreased in *rac1*-KO sWAT compared to sWAT of control mice (Figure 9). Moreover, expression levels of an array of genes that encode lipogenic enzymes, including ATP citrate lyase (ACLY), acetyl-CoA carboxylase (ACC), fatty acid synthase (FASN), stearoyl-CoA desaturase 1 (SCD1), and glycerol-3-phosphate acyltransferase (GPAT), were examined [35]. mRNA levels of these lipogenic enzymes were in fact reduced in *rac1*-KO sWAT (Figure 9).

## 3. Discussion

In an attempt to investigate the role of Rac1 in various physiological functions of adipocytes, we generated mice in which Rac1 was lacking specifically in adipocytes, named adipo-*rac1*-KO mice. A prominent phenotype that adipo-*rac1*-KO mice exhibit is atrophy of eWAT and sWAT, which is associated with a disease termed lipodystrophy in humans [36]. Lipodystrophy is characterized by a complete or partial loss of adipose tissue, causing numerous metabolic complications [36]. Lipodystrophy can be acquired or inherited. Acquired lipodystrophy is linked to infections, autoimmune diseases, and panniculitis, etc [36]. On the other hand, various mutations that cause lipodystrophy are reported [36]. For example, one of the most severe types of congenital generalized lipodystrophy is ascribed to loss-of-function mutations of the Berardinelli-Seip congenital lipodystrophy 2 gene, which is upregulated during adipogenesis and abundantly expressed in adipose tissue [37].

Mutations in *PI3K* and *akt2* genes have also been implicated in lipodystrophy in humans. A heterozygous missense mutation in the gene encoding the p85α regulatory subunit of PI3K was identified as the genetic cause of SHORT syndrome with partial lipodystrophy [38]. In addition, a missense mutation in the *akt2* gene, which renders the Akt2 protein dominant-negative, was identified in a family that showed dominant inheritance of severe insulin-resistant diabetes, and the proband of this family had partial lipodystrophy [39]. Conversely, the same de novo activating mutation in the *akt2* gene was carried by three unrelated patients with severe fasting hypoglycemia and asymmetric overgrowth (increased truncal adipose tissue) [40]. These findings support the notion that PI3K and Akt2 may be involved in the regulation of adipogenesis and hypertrophy of adipose tissue, although mutations in the *akt2* gene are thought to be uncommon causes of type 2 diabetes [41]. Consistent with the above observations in humans, Akt2-null mice exhibited a growth deficiency and an age-dependent loss of adipose tissue (lipoatrophy) [42].

Here, we report atrophy of eWAT and sWAT in adipo-*rac1*-KO mice. Furthermore, white adipocytes in these mice were smaller than those in control mice. Considering our recent findings, which provided evidence that Rac1 acts as a regulator of insulin signaling [23,24,26], it is reasonable to assume that defects in Rac1-mediated insulin action may be responsible for the impaired development of WAT in adipo-*rac1*-KO mice. A similar reduction in size of WATs observed in Akt2-null mice [42] further supports this idea, because Rac1 acts downstream of Akt2 in adipocyte insulin signaling [23,24,26].

An important role of insulin in adipocytes is stimulation of glucose uptake from the blood circulation. Our previous study, by the use of in vitro differentiated 3T3-L1 adipocytes, demonstrated that Rac1 was involved in the regulation of insulin-stimulated GLUT4 translocation to the plasma membrane [24,26]. Therefore, in this study, we further investigated the role of Rac1 in insulin-stimulated glucose uptake in white adipocytes isolated from adipo-*rac1*-KO mice. As expected, insulin-stimulated glucose uptake was totally inhibited in white adipocytes lacking Rac1 (Figure 3). In accordance, cell surface translocation of GLUT4 following insulin stimulation was markedly reduced in *rac1*-KO mouse white adipocytes (Figure 4). Given that glucose is utilized for the de novo synthesis of triacylglycerol, it is likely that defects in glucose uptake in response to insulin may be a cause for the impaired development of *rac1*-KO mouse white adipocytes and consequent atrophy of WATs. In contrast to our present results, Marcusohn et al. argued against the role of Rac1 downstream of PI3K in adipocyte insulin signaling [27]. This conclusion was based on the observation that insulin-stimulated glucose uptake was not inhibited by a dominant-negative mutant of Rac1 in 3T3-L1 adipocytes. Although the reasons for this discrepancy remain obscure, it is possible that suppression of Rac1 by the dominant-negative mutant might be insufficient [27].

FLJ00068 was identified as a GEF that directly regulated Rac1 in skeletal muscle insulin signaling [16,19,25,43]. However, the GEF responsible for the regulation of Rac1 in white adipocytes remained unclear. Thus, we tested the effect of ectopic expression of a constitutively activated mutant of FLJ00068 in mouse white adipocytes. This mutant indeed activated Rac1 and RalA (Figure 5A,B and Figure 7) and stimulated GLUT4 translocation in a manner dependent on both Rac1 and RalA (Figure 4 and Figure 6). These results are highly consistent with our previous results obtained from experiments by the use of in vitro differentiated 3T3-L1 adipocytes [26]. Collectively, it is plausible that FLJ00068 acts as a direct regulator of Rac1, not only in skeletal muscle but also in white adipocytes.

The Ras family small GTPase RalA is also implicated as a switch of insulin signaling in adipocytes [23,31]. Actually, RalA was activated following insulin stimulation in 3T3-L1 adipocytes and mouse white adipocytes [23,31]. When activated, RalA in GLUT4-containing vesicles bound to the exocyst complex, tethering GLUT4 vesicles to the plasma membrane [31]. The activation of RalA also occurred in mouse skeletal muscle in response to in vivo insulin stimulation in a Rac1-dependent manner [33]. Therefore, it is likely that RalA is involved in skeletal muscle insulin signaling, acting as a regulator of glucose uptake downstream of Rac1. However, it remains unclear whether RalA functions downstream of Rac1 in insulin signaling in adipocytes. In this study, we present in vivo evidence that RalA acts downstream of Rac1 in mouse white adipocytes (Figure 6 and Figure 8), and these results are consistent with our recent observation in in vitro differentiated 3T3-L1 adipocytes [24]. The mechanisms underlying Rac1-dependent RalA activation in response to insulin in white adipocytes need to be elucidated in future studies.

Rac1 regulates cortical actin remodeling, which is a prerequisite for GLUT4 vesicle transport [5,6]. Additionally, RalA-mediated tethering of GLUT4-containing vesicles to the plasma membrane may be regulated by Rac1 (Figure 6 and Figure 8) [31]. On the other hand, insulin-stimulated phosphorylation of the GAP AS160 by Akt2 attenuated its GAP activity, leading to the activation of the Rab family member Rab10 in adipocytes [5,6,7,9,10]. Activated Rab10 in turn induced GLUT4 translocation [9,10]. It remains unclear whether the regulation of Rab10 by AS160 is affected by the deficiency of Rac1, and future studies are required to clarify the role of Rac1 in the regulation of Rab proteins in adipocytes.

Herein, we propose that failure of insulin-stimulated glucose uptake in Rac1-deficient white adipocytes may cause their reduced size and atrophy of WATs. In addition, we show that mRNA levels of enzymes that are involved in the de novo synthesis of lipids are decreased in *rac1*-KO sWAT. This may be another explanation for atrophy of WATs. Of course, we do not exclude the possibility that Rac1 may also be required for other insulin-stimulated processes in the development of white adipocytes. For instance, it is possible that Rac1 may play an important role in insulin-stimulated transport of fatty acids into white adipocytes, because mechanisms for insulin-dependent cell surface translocation of fatty acid transporters are similar to those for GLUT4 translocation [44]. Moreover, Rac1 may regulate expression of a variety of genes, such as those required for the maturation of WATs and the degradation of stored lipids. These possibilities will be investigated in our laboratory in the future.

We show that adipo-*rac1*-KO mice develop whole-body glucose intolerance and insu-lin resistance (Figure 1E,F). Defects in insulin-dependent glucose uptake in *rac1*-KO WAT may be a cause of these phenotypes. However, it is plausible that other mechanisms for inducing whole-body glucose intolerance and insulin resistance in adipo-*rac1*-KO mice may exist, considering that the contribution of WAT to insulin-dependent alteration in the blood glucose level is not so large. For instance, it is possible that atrophy of WAT may negatively affect insulin-stimulated glucose uptake in skeletal muscle due to a decrease in the secretion of some adipokines, such as adiponectin. Compensatory mechanisms (increased insulin sensitivity) in WAT are indeed reported in muscle-specific *rac1* KO mice on a high-fat diet, supporting the existence of tissue cross-talk between WAT and skeletal muscle [45].

## 4. Materials and Methods

### 4.1. Materials

A rat monoclonal antibody against the HA epitope tag (11 867 423 001), a rabbit polyclonal antibody against the HA epitope tag (561), a rat monoclonal antibody against the Myc epitope tag (GTX10910), and a goat polyclonal antibody against the V5 epitope tag (A190-119A) were purchased from Roche Applied Science (Mannheim, Germany), MBL (Tokyo, Japan), GeneTex (Irvine, CA, USA), and BETHYL (Montgomery, TX, USA), respectively. Mouse monoclonal antibodies against Rac1 (610650) and RalA (610221) were purchased from BD Biosciences (San Diego, CA, USA). An anti-α-tubulin antibody (T9026) was purchased from Sigma-Aldrich (St. Louis, MO, USA). Antibodies against goat IgG, mouse IgG, rabbit IgG, and rat IgG conjugated with CF™ 350/543/647 were purchased from Biotium (Fremont, CA, USA). Antibodies against mouse IgG (NA9310) and rat IgG (NA935) conjugated with horseradish peroxidase were purchased from Cytiva (Emeryville, MA, USA). Insulin was purchased from Eli Lilly (Indianapolis, IN, USA). The Akt2-specific inhibitor AI-XII and the Rac1-specific inhibitor RI-II were purchased from Sigma-Aldrich.

### 4.2. Animal Experiments

All animal experiments were approved by the Ethics Committee for Animal Experiments at Osaka Prefecture University (Approval Code: #20-74 and #20-75; approved on 1 April 2020) and carried out according to institutional guidelines of Osaka Prefecture University. Mice on the C57BL/6 genetic background were used in this study. We routinely crossbred *rac1^flox/flox^* mice [28] with *rac1^flox/flox^*; adipoq-Cre (adipo-*rac1*-KO) mice to obtain adipo-*rac1*-KO mice for experiments. Adipoq-Cre transgenic mice [29] were used as controls throughout this study. Mice were fed a normal chow diet and adult (25- to 26-week-old) male mice were used for all in vivo and ex vivo experiments.

### 4.3. Genotyping

PCR primers for genotyping were as follows: 5′-ATTTTCTAGATTCCACTTGTGAAC-3′ and 5′-ATCCCTACTTCCTTCCAACTC-3′ for the *rac1* gene, and 5′-AGGTTCGTTCACTCATGGA-3′ and 5′-TCGACCAGTTTAGTTACCC-3’ for the Cre recombinase transgene.

### 4.4. Immunoblot Analysis

Proteins separated by SDS-polyacrylamide gel electrophoresis were transferred onto a 0.45 μm pore size polyvinylidene difluoride membrane (Cytiva). Membranes were incubated with anti-HA tag, anti-Rac1, and anti-α-tubulin antibodies, respectively, and horseradish peroxidase-conjugated secondary antibodies. Specific proteins were visualized by Chemi-Lumi One Ultra (Nacalai tesque, Kyoto, Japan). Images were captured, and densitometric analysis was carried out by using a chemiluminescence imaging system (Ez-Capture MG, Atto, Tokyo, Japan).

### 4.5. Glucose and Insulin Tolerance Tests

For glucose tolerance tests, mice were fasted for 16 h and glucose (1.5 g/kg of body weight) was administered via intraperitoneal injection. Blood samples were obtained from a cut at the tip of the tail at 0, 15, 30, 60, 90, and 120 min, and glucose levels were measured using a portable blood glucose analyzer (Glutest Neo; Sanwa Chemical, Nagoya, Japan) according to the manufacturer’s instructions. For insulin tolerance tests, mice were fasted for 6 h and insulin (175.5 µg/kg of body weight) was administered via intraperitoneal injection. Blood glucose levels were measured at the same time points as in glucose tolerance tests.

### 4.6. Histological Analysis

Formalin-fixed, paraffin-embedded WAT sections were stained with hematoxylin and eosin according to standard protocols. Images were captured with an optical microscope (CKX53, Olympus, Tokyo, Japan), and adipocyte areas were measured using ImageJ software. Values of 100 cells (in 25 images in total from 5 different mice) for each condition were used for statistical analysis (Student’s t-test).

### 4.7. Gene Transfer into White Adipocytes by Electroporation

Plasmid DNAs were introduced into mouse subcutaneous white adipocytes by electroporation. Mice were anesthetized by intraperitoneal injection of a solution of medetomidine (0.3 mg/kg of body weight), midazolam (4.0 mg/kg of body weight), and butorphanol (5.0 mg/kg of body weight). A combination of expression vectors (pCAGGS-GLUT4*myc*7-GFP, pCAGGS-Myr-p110α-HA×3, pCAGGS-Myr-Akt2-HA×3, pCAGGS-HA×2-FLJ68ΔN, pCAGGS-HA×3-Rac1(G12V), and pCAGGS-V5×3-RalA(S28N)) (80 µg in total) were dissolved in 50 µL of 9 mg/mL NaCl and injected into sWAT with a 27-gauge needle. After injection, sWAT was placed between the electrodes (tweezer type, 3 mm diameter, CUY650P3 (Nepa Gene, Chiba, Japan)), and square wave electrical pulses (30 ms) were applied twice (80 V and 72 V, respectively) at 50 ms intervals (for poring) using a pulse generator (NEPA21 Type II, Nepa Gene). Subsequently, square wave electrical pulses (30 ms) were applied seven times (30 V, 27 V, 24.3 V, 21.9 V, 19.7 V, 17.7 V, and 15.9 V, respectively) at 200 ms intervals followed by seven pulses under the same conditions, except that the polarity was the opposite (for transfer).

### 4.8. Preparation of Mature White Adipocytes from sWAT and Their Primary Cultures

sWAT was excised from euthanized adult male mice, minced with scissors, and incubated in collagenase buffer (20 mM HEPES (pH 7.4), 120 mM NaCl, 5 mM KCl, 4 mM NaHCO_3_, 1 mM CaCl_2_, 0.7 mM MgSO_4_, 0.4 mM KH_2_PO_4_, 0.3 mM Na_2_HPO_4_, and 4 mg/mL collagenase I (031-17601, Fujifilm Wako, Osaka, Japan)) at 37 °C for 1 h. Subsequently, the floating layer containing mature adipocytes was collected. Isolated mature adipocytes were then resuspended and cultivated in Dulbecco’s modified Eagle’s medium (DMEM) supplemented with 10% (*v*/*v*) fetal bovine serum, 1 mM sodium pyruvate, 500 IU/mL penicillin, and 500 μg/mL streptomycin.

### 4.9. Measurement of the Uptake of 2-DG

Primary cultured mature white adipocytes from control and adipo-*rac1*-KO mice were plated at a density of 2 × 10^4^ cells per well in a 96-well cell culture plate. Cells were starved in glucose-free DMEM (A1443001, Thermo Fisher Scientific, Waltham, MA, USA) for 3 h prior to stimulation with 100 nM insulin for 30 min. Cells were then incubated with 1 mM 2-DG for 15 min, and the uptake of 2-DG was measured using the Glucose Uptake-Glo assay kit (Promega (Madison, WI, USA)) according to the manufacturer’s instructions. The luminescence signal was measured using a multi-detection microplate reader (Synergy HT, BioTek (Winooski, VT, USA)).

### 4.10. Detection of GLUT4 Translocation to the Plasma Membrane by a Reporter Assay

Mice were fasted for 16 h, and insulin (175.5 µg/kg of body weight) was administered via intravenous injection. Forty five minutes later, mice were euthanized, and sWAT was ablated from these mice. Isolated sWAT was then fixed with 40 mg/mL paraformaldehyde in phosphate-buffered saline (PBS) for 30 min and was incubated with an anti-Myc tag antibody overnight at 4 °C for the detection of plasma membrane translocation of the GLUT4 reporter GLUT4*myc*7-GFP [30]. After washing three times with PBS, sWAT was fixed again with 40 mg/mL paraformaldehyde in PBS for 10 min, permeabilized with 0.5% (*v*/*v*) Triton X-100 in PBS for 15 min, and incubated in 0.1% (*v*/*v*) Triton X-100 in PBS supplemented with 2% (*v*/*v*) normal goat serum (G9023, Sigma-Aldrich) for 30 min. Permeabilized cells were further treated with anti-HA (for the detection of ectopically expressed Myr-p110α, Myr-Akt2, FLJ68ΔN, or Rac1(G12V)), anti-Rac1 (for the detection of endogenous Rac1), or anti-V5 (for the detection of ectopically expressed RalA(S28N)) antibodies for 2 h. After washing three times with 0.1% (*v*/*v*) Tween 20 in PBS, anti-Myc, anti-HA, anti-Rac1, and anti-V5 antibodies were detected with fluoresceinated secondary antibodies. Images were obtained and analyzed using a confocal laser-scanning microscope (FV1200, Olympus). Fluorescent intensities of Myc and GFP in regions of interest were quantified using ImageJ software. The relative amount of GLUT4*myc*7-GFP translocated to the plasma membrane was estimated by the ratio of Myc and GFP fluorescent intensities (Myc/GFP). Values of 15 cells in total from 5 different images for each condition were used for statistical analysis (Student’s t-test).

### 4.11. Detection of the Activation of Rac1 and RalA

Mice were fasted for 16 h, and insulin (175.5 µg/kg body weight) was administered via intravenous injection. Forty five minutes later, mice were euthanized, and sWAT was ablated from these mice. In some experiments, sWAT was treated with 5 μM AI-XII or 25 μM RI-II for 2 h, and then stimulated ex vivo with 100 nM insulin for 45 min instead of being stimulated via intravenous injection. sWAT was fixed in overlay assay buffer (50 mM HEPES-NaOH (pH 7.3), 150 mM NaCl, 20 mM MgCl_2_, and 0.05% (*v*/*v*) Tween 20) supplemented with 20 mg/mL paraformaldehyde on ice for 30 min. Fixed sWAT was washed three times with overlay assay buffer and incubated with GST-POSH(251–489)-V5×3 (for the activated form of Rac1, 400 μg/mL) or GST-V5×3-Sec5(1–99) (for the activated form of RalA, 400 μg/mL) in overlay assay buffer supplemented with 0.1% (v/v) Triton X-100 and 50 μg/mL bovine serum albumin on ice for 1 h. GST-POSH(251–489)-V5×3 and GST-V5×3-Sec5(1–99) were purified from *Escherichia coli* transformants as previously described [16]. After washing three times with overlay assay buffer, sWAT was fixed again in overlay assay buffer supplemented with 20 mg/mL paraformaldehyde on ice for 20 min. Fixed sWAT was washed three times with 0.1% (*v*/*v*) Tween 20 in PBS and incubated with an antibody against the V5 tag for the detection of GST-POSH(251–489)-V5×3 or GST-V5×3-Sec5(1–99). sWAT was counterstained with an antibody against Rac1 or RalA for the estimation of the total amount of Rac1 or RalA. Ectopically expressed Myr-p110α, Myr-Akt2, FLJ68ΔN, and Rac1(G12V) were detected by an anti-HA tag antibody. Anti-V5, anti-HA, anti-Rac1, and anti-RalA antibodies were detected by fluoresceinated secondary antibodies. Images were obtained and analyzed using a confocal laser-scanning microscope (FV1200, Olympus). Intensities of fluorescent signals of V5 and Rac1 or RalA in regions of interest were quantified using ImageJ software. The activity of Rac1 or RalA was estimated by the ratio of fluorescence signal intensities (V5/Rac1 or V5/RalA). Values of 15 cells in total from 5 different images for each condition were used for statistical analysis (Student’s t-test).

### 4.12. Quantitative RT-PCR Analysis

The total cellular RNA was isolated from sWAT using Sepasol-RNA I Super G (Nacalai tesque, Japan), according to the manufacturer’s instructions. cDNAs were synthesized using the SuperScript IV first-strand synthesis system for RT-PCR (Termo Fisher Scientific). PCR was carried out using TB Green Premix Ex Taq II (Takara Bio (Kyoto, Japan)) and specific primers (Termo Fisher Scientific) with Thermal Cycler Dice Real Time System III (Takara Bio) according to the manufacturer’s instructions. PCR primers were as follows: 5′-AGCATCAGGCTTCCACTATG-3′ and 5′-TGGATCCGGCAGTTAAGATC-3′ for the *pparg* gene, 5′-GTCTACATCCTTGACTTGGC-3′ and 5′-CACTTTTGGCATCCAGGTCT-3′ for the *acly* gene, 5′-TACCTGTACAAGCAGTGTGG-3′ and 5′-CAATCCACTCGAAGACCACT-3′ for the *acc* gene, 5′-TTGCTGGCACTACAGAATGC-3′ and 5′-CTCAGAGCGACAATATCCAC-3′ for the *fasn* gene, 5′-GAGTACGTCTGGAGGAACAT-3′ and 5′-AGAGCGCTGGTCATGTAGTA-3′ for the *scd1* gene, 5′-GCTGGGTGTTACTAAAGCTC-3′ and 5′-GTCAATGTGGGATCTGTGCA-3′ for the *gpat* gene, and 5′-ATGAAGATCAAGATCATTGCTCCTC-3′ and 5′-ACATCTGCTGGAAGGTGGACAG-3′ for the β-actin gene. Relative mRNA levels were determined by the ΔΔCt method followed by normalization with the β-actin mRNA level.

## Data Availability

The data presented in this study are available on request from the corresponding author.

## Abbreviations

2-DG	2-deoxy-D-glucose
ACC	acetyl-CoA carboxylase
ACLY	ATP citrate lyase
adipo-*rac1*-KO	adipocyte-specific *rac1* knockout
BAT	brown adipose tissue
ctrl	control
DMEM	Dulbecco’s modified Eagle’s medium
eWAT	epididymal white adipose tissue
FASN	fatty acid synthase
GAP	GTPase-activating protein
GEF	guanine nucleotide exchange factor
GFP	green fluorescent protein
GPAT	glycerol-3-phosphate acyltransferase
GST	glutathione *S*-transferase
HA	hemagglutinin
Myr-Akt2	an N-terminally myristoylated form of Akt2
Myr-p110α	an N-terminally myristoylated form of the phosphoinositide 3-kinase catalytic subunit p110α
PBS	phosphate-buffered saline
PCR	polymerase chain reaction
PI3K	phosphoinositide 3-kinase
PPARγ	peroxisome proliferator-activated receptor γ
RT	reverse transcriptase
SCD1	stearoyl-CoA desaturase 1
sWAT	subcutaneous white adipose tissue
WAT	white adipose tissue
